# Design and Experiments of a Piezoelectric Motor Using Three Rotating Mode Actuators

**DOI:** 10.3390/s19235184

**Published:** 2019-11-26

**Authors:** Roland Ryndzionek, Łukasz Sienkiewicz, Michał Michna, Filip Kutt

**Affiliations:** Gdańsk University of Technology, Faculty of Electrical and Control Engineering, 80-233 Gdańsk, Poland; lukasz.sienkiewicz@pg.edu.pl (Ł.S.);

**Keywords:** piezoelectric actuator, piezoelectric ultrasonic motor, finite element analysis, piezoelectricity

## Abstract

This paper represents a numerical and experimental investigation of the multicell piezoelectric motor. The proposed design consists of three individual cells that are integrated into the stator, double rotor, and a preload system combined into a symmetrical structure of the motor. Each of the cells is characterized by a traveling wave and rotating mode motor. A finite element numerical analysis is carried out to obtain optimal geometrical dimensions of the individual cell in terms of generated vibrations and resonant frequencies of the structure. The results of the numerical analysis are compared with analytical calculations based on the equivalent circuit theory. Experimental tests are also presented, including laser interferometry measurements of vibrations generated at the surface of the stator, impedance analysis, as well as measurements of mechanical characteristics of the complete motor. The final stage of the study concludes that the presented motor can provide relatively high torque compared with other traveling wave rotary motors.

## 1. Introduction

The subject of piezoelectric motors, sensors, and actuators has been, and still is widely discussed in the literature. Nevertheless, there is still great potential for the development of new structures and the improvement of existing ones. Some of the solutions have been commercialized and are used in the industry. The first invented and registered piezoelectric motor appeared in the USA in the 1940s [1]. In the following years, the great development of this technology could be observed. The industry acknowledged the enormous potential of the technology and invested huge amounts of resources on practical developments during the 1980s. Many new devices were introduced, including precision positioners with high strain materials, mass-produced multilayer devices for portable electronic appliances, ultrasonic motors for micro-robotics, and smart structures [2,3,4]. Economic stagnation and crisis in the 1990s slowed down the development of this technology. The exploration of new active materials slowed down as well. However, a multitude of new typologies and solutions have already been proposed in the twenty-first century [5,6,7]. In [3], Kenji Uchino wrote that we are currently experiencing a “*renaissance*” of piezoelectric actuators.

The first research work on piezoelectricity was done by Carl Linnaeus and Franz Aepinus in the mid-18th century [8]. Their experiments included testing various materials, and examination of a voltage generated as an effect of temperature variations. This phenomenon was named pyroelectricity. Continuing that study, both René Just Haüy and Antoine César Becquerel posited a connection between mechanical stress and electric charge in some materials [9,10]. The term “piezoelectricity”, introduced by Wilhelm Gottlieb Hankel, describes the phenomenon of the induction of an electric charge or voltage in response to applied mechanical force or pressure to a piezoelectric material. Conversely, if some charge or voltage is imposed on a piezoelectric material, the material reacts by generating some mechanical force and strain [11]. This phenomenon is called the inverse piezoelectric effect.

The direct piezoelectric effect was confirmed experimentally by Pierre and Jacques Curie in 1881. A year later, the Curie brothers demonstrated the existence of an inverse effect based on mathematical considerations and the thermodynamic research of Gabriel Lippmann [12,13,14,15].

Materials that exhibit a significant and useful piezoelectric effect fall into three main groups: natural and synthetic crystals, polarized piezoelectric ceramics, and certain polymer films. Ferroelectric ceramics are the most common piezoelectric material in today’s engineering applications. Among them, polycrystalline ceramics like barium titanate (BaTiO${}_{3}$) and lead zirconate titanate (PZT) are the most popular materials, in particular due to its low manufacturing costs and almost arbitrary shaping possibilities compared to single crystalline piezoelectrics.

Piezoelectric ceramics are usually divided into two groups. The antonyms “hard” and “soft” doped piezoelectric materials refer to the ferroelectric properties, that is, the mobility of the dipoles or domains, and hence also to the polarization/depolarization behaviour. “Hard” piezoelectric materials are those materials whose properties are stable with temperature, electric field, and stress. They are used in applications requiring high power actuation or projection. The applications often have a narrow bandwidth, but are usually operated either at resonance or well under resonance. “Soft” piezoelectric materials are those materials whose properties have been enhanced for sensing, actuation, or both. They have high coupling and high permittivity. Property enhancement was made at the expense of the temperature, electric field, and stress stability [16].

**Figure 1:**
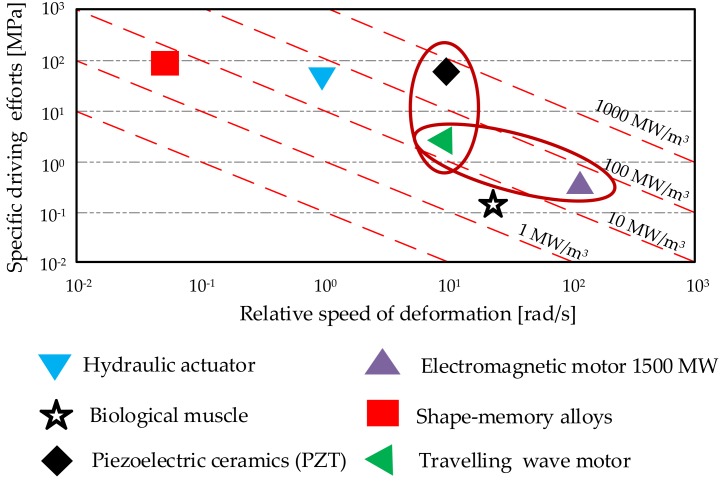
A comparison of transducer technologies [19,20]. Maximum actuation force and velocity.

Among the existing piezoelectric materials, hard PZT ceramics have the highest ability for application in the field of actuators, motors, or motion stages [17,18]. The comparison of chosen electromechanical transducer technologies and materials is introduced in Figure 1. In the figure, the Y-axis describes specific driving efforts of transducers. This is the ability to produce the effort in terms of volume. The X-axis describes the relative speed of deformation. It represents the speed at which the transducer’s active part can be deformed and go back to its bulk. The product of these two quantities gives the theoretical power density. There is an observable distance in terms of specific driving efforts between the traveling wave motor and PZT ceramics. It clearly shows the remaining potential to be extracted from this electro-active material by novel actuator and motor designs.

### 1.1. Classification of the Proposed Motor

**Figure 2:**
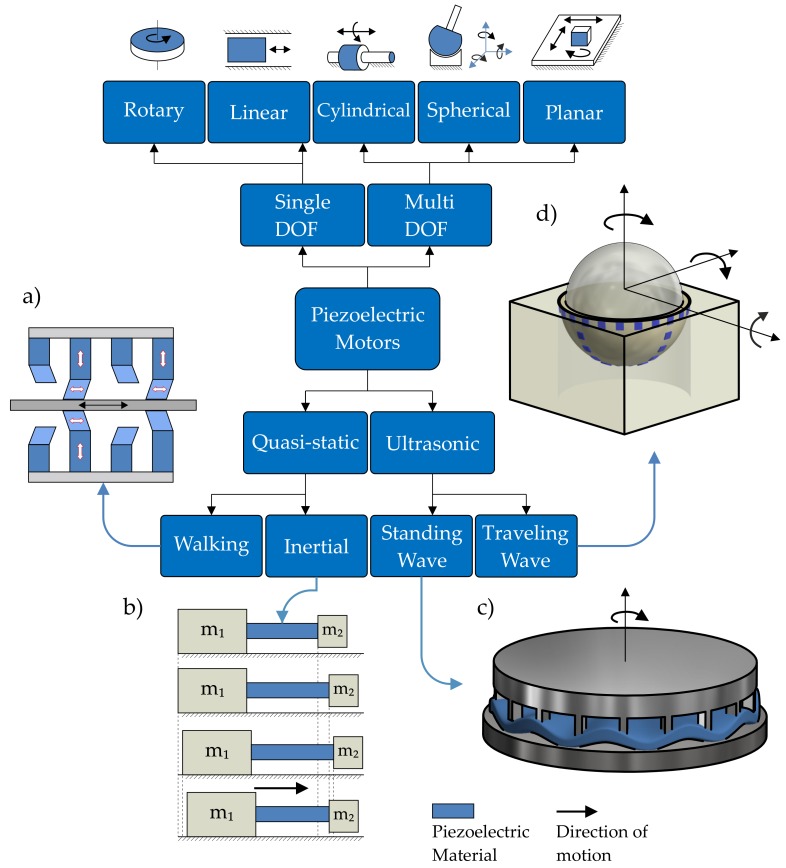
A classification of piezoelectric motors according to vibration types and to output motion: (**a**) walking linear motor [28]; (**b**) inertial linear motor [19]; (**c**) standing wave rotary motor [31]; (**d**) traveling wave spherical motor [29].

The two most common classification methods of piezoelectric motors are based on either the vibration type generated by the inverse piezoelectric phenomenon, or the output motion produced by a motor (Figure 2). The widest group of piezoelectric motors in terms of vibration generation is resonant or ultrasonic motors [21,22]. An ultrasonic piezoelectric motor is one in which electrical energy is converted by the inverse piezo-effect to obtain displacement of the actuator at one of its resonant frequencies in the ultrasonic range. The displacements of the actuator results from excitation of different acoustic waves in the actuator shape. The waves generated by the actuator can be standing or traveling waves, depending on the number of contact points between the stationary and the moving part. On one hand, the use of standing waves (one point of contact) leads to a simpler mechanical structure of the motor and more basic driving circuitry, but on the other hand, results in limited output power [23]. Traveling wave motors generally have a larger power output, though at the expense of a more complex mechanical design.

The motors operating outside resonance conditions fall into the group of quasi-static designs. Inertial quasi-static designs use the high and low speed profile of displacement, creating inertial motions of the motor. Structures using the inertial actuation mode can be designed as simple mechanical assemblies and driven with a single electrical source. This contributes to the notion that piezoelectric motors often have simpler and less complicated control systems than comparable electro-magnetic motors which do not require complex control strategies or a controller-observer approach [24,25,26,27].

Quasi-static walking motors use alternating motions of clamp and feed to advance the moving part step-by-step. The operation principle of this group was inspired by motions found in nature. The actuators using the walking mode have long strokes and high resolutions, but the whole motor structure and excitation signals are complex, due to the multiple groups of piezoelectric actuators [28].

Classification of piezoelectric motors based on the characteristics of the output motion describes the number of directions which motion piezoelectric structures can produce. The most common rotary and linear motors are single degree-of-freedom (DOF) designs. More complex structures are often preferable in micromechatronics systems, where multi-DOF motors are the optimal solution for reducing weight and volume [29]. Taking the above classifications into account (Figure 2), the presented design is a single-DOF, rotary, ultrasonic, traveling-wave piezoelectric motor.

### 1.2. Description of the Proposed Motor

The purpose of this effort was to develop a structure which will combine the topology of the traveling wave and rotating-mode actuators. Moreover, it will work in the ultrasonic range (above 20 kHz). The motor was designed for embedded actuation in aircraft and automotive applications. Its compact structure and a simple principle of operation enable use-cases where traditional motors will not be optimal.

**Figure 3:**
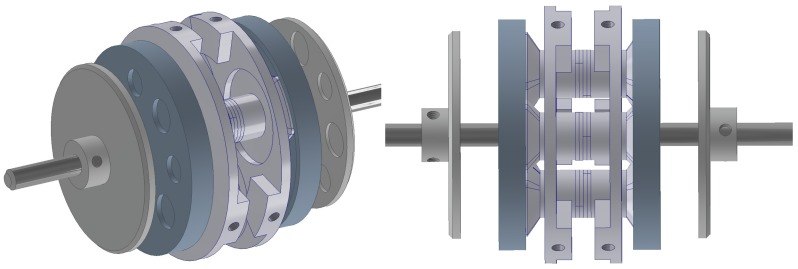
The structure of MPM.

The structure is referred to as a Multicell Piezoeletric Motor (MPM) (Figure 3). The phrase “multicell” has been used because the topology for each rotating-mode actuator can be considered as an independent structure—referred to as a “single cell”. The MPM has the following parts: stator, rotors, piezoelectric ceramic stacks, and shaft. The stator consists of two pairs of piezoceramics and countermasses with three rotating-mode actuators. A proper mix of the performance of the three rotating-mode actuators will generate three traveling waves. Selected analysis results and experimental tests of the MPM have been described in [30]. The authors present the continuation of the research in MPM development.

The paper is organized as follows. In Section 2, analytical models of piezoelectric structures are described and analysed. In Section 3, detailed design and Finite Element Analyses are investigated and discussed. Performance of the test experiments are described in Section 4. Finally, the paper summarizes this effort with the main conclusions.

## 2. Analytical Models of Piezoelectric Transducers

**Figure 4:**
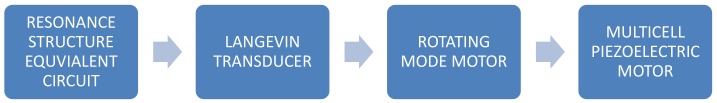
The stages of analytical modeling of the resonance structures and MPM.

In this section, analytical modeling of the basic structures of piezoelectric motors will be briefly described. The discussion of specific circuits is important in understanding the MPM operation principle. The structure is strongly based on these equivalent circuits. Firstly, the modeling of the resonance structure using the Mason’s equivalent circuit is presented. Next, the principles and basic relationships of Langevin’s transducer and rotating-mode motor are described. The structure of the MPM is based on three rotating-mode piezoelectric motors. Since the rotating-mode motor is based on Langevin’s transducer, a brief description and analysis of the resonance structures using the analytical modeling approach are presented, as shown in Figure 4. Crucial physical quantities used in this analysis are explained in Table 1:

### 2.1. Simple Equivalent Circuit

**Figure 5:**
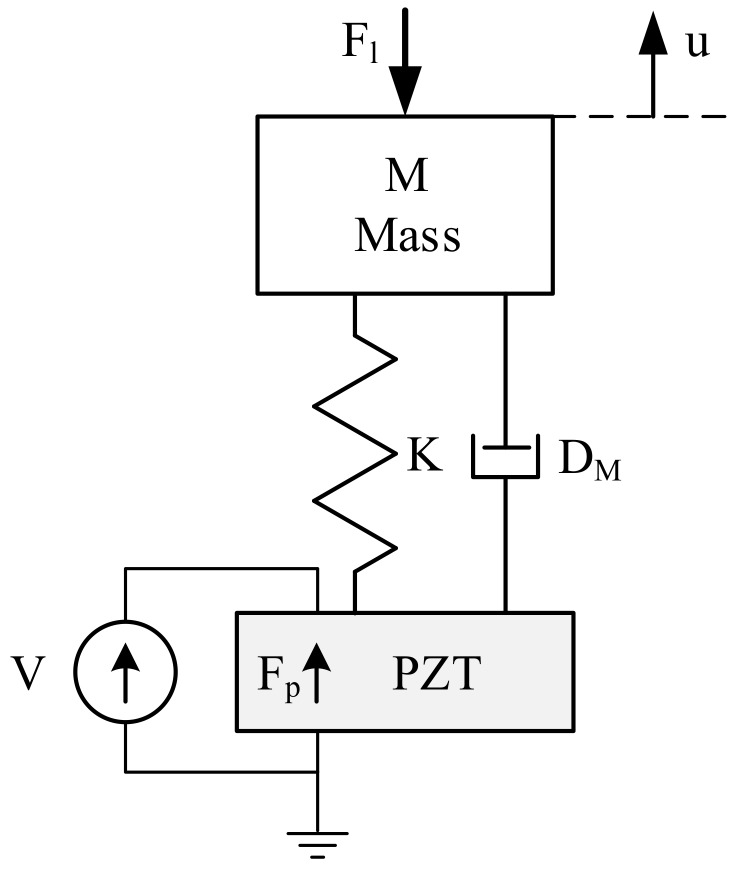
Model of the elementary piezoceramic resonance system.

Mathematical models of piezoelectric transducers are an important tool supporting the design process. Depending on the design stage, mathematical models with varying degrees of detail are required [32,33]. Analysis of the simulation results can be used to select an appropriate active and passive material, as well as plate and counter-mass dimensions. The simplest model of an elementary piezoceramic resonance system is presented in Figure 5. It has the following parts: piezoceramic denoted by *PZT*, counter-mass denoted by *M*, screw spring constant denoted by *K*, damping factor denoted by $D_{M}$, voltage source denoted by *V*, and force load denoted by $F_{l}$. Since the mass of the piezoelectric ceramic is negligible compared to mass *M*, its kinetic energy can be assumed as negligible.

The mechanical system shown in Figure 5 can be described by the equation:

(1) $$F_{p}\left(t\right)=M\cdot \ddot{u}\left(t\right)+D_{M}\cdot \dot{u}\left(t\right)+K\cdot u\left(t\right)+F_{l}\left(t\right)$$

where u(t) is the mass position, $F_{p}\left(t\right)$ is the piezoelectric force, $F_{l}\left(t\right)$ is the load force, *M* is the mass, $D_{M}$ is the damping factor, and *K* is the screw spring constant.

**Figure 6:**
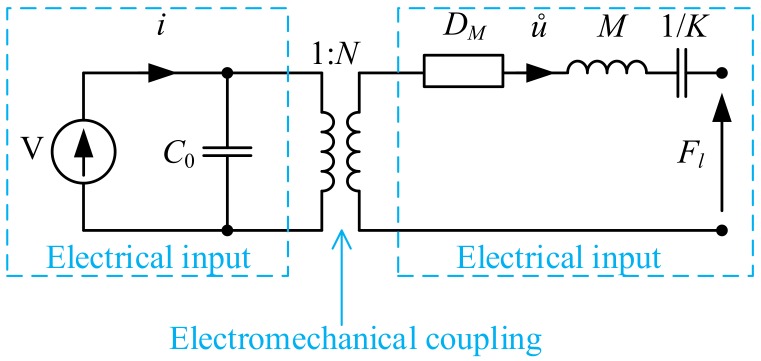
Electromechanical equivalent circuit of the piezoelectric resonance system: *L* is the inductance, electrical equivalent of mass; *C* is the capacitance, electrical equivalent of compliance; and *R* is the resistance, electrical equivalent of damping.

The equivalent electrical circuit of the mechanical system was developed based on analogies between the electrical and mechanical quantities. According to this approach, the complete representation of the piezoelectric transducer (Figure 5) in terms of the electrical variables is presented in Figure 6 and referred to as Mason’s model. The piezoelectric ceramic effect (electromechanical coupling) is modeled by an ideal transformer (with ratio *N*). The blocking capacity $C_{0}$ is the capacitance of the piezoelectric ceramic at zero strain. This simplified model is useful for analyzing the transducer operation at a frequency close to resonance frequency. It can be used for the pre-selection of counter-mass dimensions.

### 2.2. Wave Propagation in Resonance Structure

**Figure 7:**
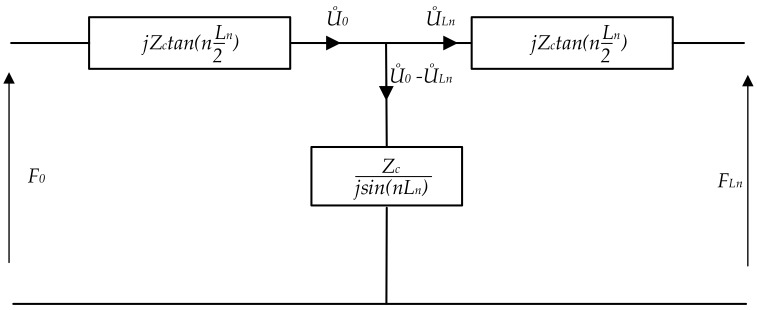
Equivalent circuit of the non-piezoelectric bar: $Z_{C}$, $L_{N}$, and *n* denote characteristic impedance of the elastic bar, length of the bar, and wave number, respectively.

In order to derive a more accurate model, the mechanical wave propagation in the transducer is analyzed. It begins with the assumption that the piezoelectric actuator is a non-piezoelectric bar of finite length. The Mason model of this structure in the form of a circuit diagram is presented in Figure 7. The waveform is not unidirectional, and a reflection of the component could happen. The wave propagation is described by the equation:

(2) $$\begin{array}{c} \dot{u}\left(x\right)=j\omega \left(\alpha^{-jnx}+\beta^{jnx}\right) \end{array}$$

where the coefficients α and β depend on the boundary condition, and $n^{2}=\omega^{2}\rho /c$.

The wave equations at both bar ends x=0 and $x=L_{n}$ are respectively described by:

(3) $$\begin{array}{c} {\dot{U}}_{0}=\dot{u}\left(0\right)=j\omega \left(\alpha +\beta \right)\\ {\dot{U}}_{Ln}=\dot{u}\left(L_{n}\right)=j\omega \left(\alpha e^{-jnL_{n}}+\beta e^{+jnL_{n}}\right) \end{array}$$

Coefficients α and β can be calculated using the above equation. Then, forces $F_{0}$ and $F_{Ln}$ at the respective extremities of the bar can be written as:

(4) $$\left\{\begin{array}{c} F_{0}=F\left(0\right)=Z_{c}\left(\frac{{\dot{U}}_{0}-{\dot{U}}_{ln}}{j\sin\left(nL_{n}\right)}+j{\dot{U}}_{0}\tan\left(n\frac{L_{n}}{2}\right)\right)\\ F_{Ln}=F\left(L_{n}\right)=Z_{c}\left(\frac{{\dot{U}}_{0}-{\dot{U}}_{ln}}{j\sin\left(nL_{n}\right)}+j{\dot{U}}_{ln}\tan\left(n\frac{L_{n}}{2}\right)\right) \end{array}\right\}$$

**Figure 8:**
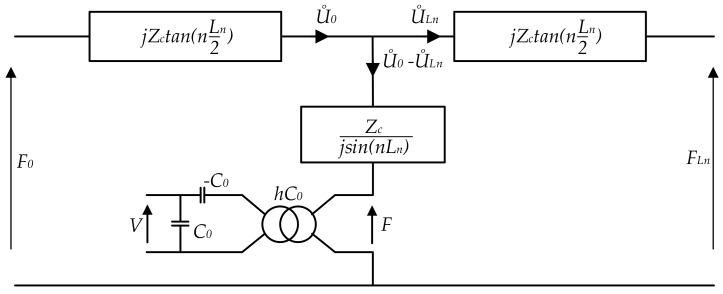
The overall Mason’s model—the electrical equivalent circuit of the structure with an added electromechanical transformer and $hC_{0}$ ratio (modeling the piezoelectric element).

An equivalent circuit diagram for the piezoelectric bar is obtained by supplementing the non-piezoelectric element with a transformer modeling the piezoelectric ceramics (Figure 8). For this case, the piezoelectric equations that describe the linear nature of piezoelectricity are used. The piezoelectric coefficients are described by the following relationships:

(5) $$\begin{array}{c} S^{D}=S^{E}-d^{t}\beta^{S}d\\ g=\beta^{T}f\\ \beta =\frac{1}{\epsilon }\\ h=\beta^{S}cd \end{array}$$

The mechanical strain in the piezoceramic material is expressed by *S* that is added to dielectric displacement *D*. These piezoelectric coefficients are used to determine the longitudinal mode of coupling. For this type of deformation, the electric field *E* and stress deformation *T* are collinear and could interact (but *E* on the ceramics surface may not be constant). On the other hand, for a ceramic without the electrical charge, the ∇D is 0; therefore *D* is a constant on the entire length of the ceramic. Taking stress *T* into account, Equation (5) can be transformed as follows:

(6) $$T=c^{D}S-hD⟶T+hD=c^{D}S$$

where

(7) $$\begin{array}{c} c^{D}=C^{E}+\frac{e^{2}}{\epsilon^{S}}\\ h=\frac{e}{\epsilon^{S}} \end{array}.$$

The substitution of Equation (6) into (4) yields the following expressions relating the mechanical parameters:

at x=0:

(8) $$F_{0}+hDA=Z_{c}\left(\frac{{\dot{U}}_{0}-{\dot{U}}_{Ln}}{j\sin\left(nL_{n}\right)}+j{\dot{U}}_{0}\tan\left(\frac{nL_{n}}{2}\right)\right)$$

at $x=L_{n}$:

(9) $$F_{Ln}+hDA=Z_{c}\left(\frac{{\dot{U}}_{0}-{\dot{U}}_{Ln}}{j\sin\left(nL_{n}\right)}+j{\dot{U}}_{Ln}\tan\left(\frac{nL_{n}}{2}\right)\right)$$

### 2.3. Rotating Mode Motor

**Figure 9:**
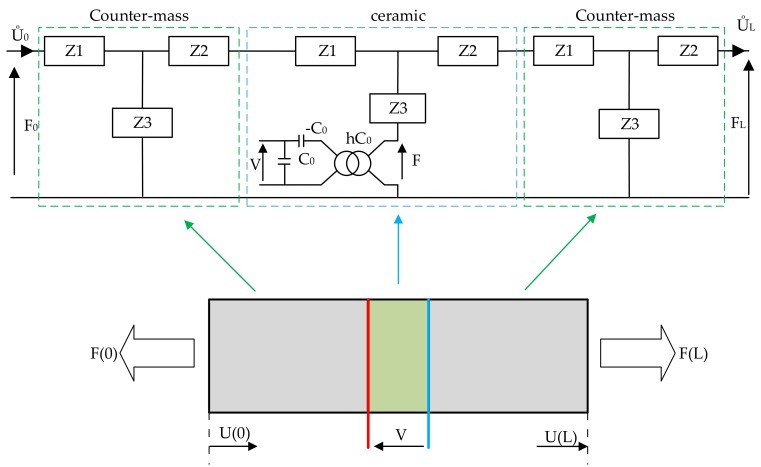
The overall diagram of Langevin’s transducer.

A brief model description of the piezoelectric bar is considered as an introduction to modeling the rotating-mode motor. The next stage in modeling is Langevin’s transducer. The transducer has a simple structure: two counter-masses made of aluminum, and one or more piezoelectric ceramics. Each element of Langevin’s transducer may be represented by an overall equivalent circuit diagram (Figure 9). The ends of the transducer are not loaded, thus there are no constrains —this is represented by a short circuit on the diagram. The piezoelectric ceramics are supplied and therefore deformed at a frequency corresponding to the mechanical resonance frequency of the structure. Therefore, the initial deformation of ceramics is amplified by the effects of the resonance of the mechanical structure.

The rotating-mode motor consists of the same parts as a Langevin-type transducer, which is basically composed of one or more pairs of piezoceramic rings sandwiched between two metal counter-masses (stator). The difference is in the excited mode: in the rotation-mode motor, the mode of flexion exists, whereas in the Langevin-type transducer, longitudinal vibration modes exist. The two sinusoidal, high-frequency, phase-shifted voltage sources are used to supply the ceramics’ structures. The ceramics are oriented in reference to each other and shifted by 90 degrees. This allows for the travelling wave to be generated.

**Figure 10:**
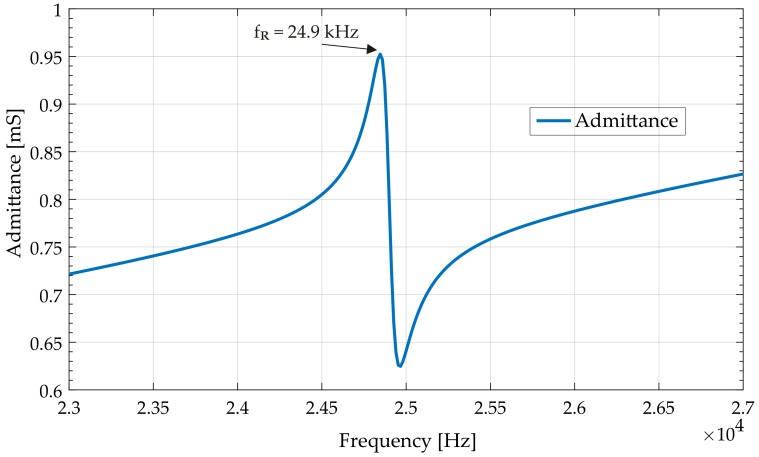
Admittance of the individual actuator calculated with the analytical model.

The analytical model of the rotating-mode motor was validated using admittance characteristics (Figure 10). The following parameters of the structure were used: the diameter of an actuator 12.5 mm, external diameter of PZT 12.5 mm; internal diameter of PZT 5 mm; and thickness 1 mm. The length of the counter-masses was 15 mm. The obtained frequency of resonance was $f_{R}$ = 24.9 kHz compared to 22.57 kHz simulated in modal finite element analysis. Differences can mostly be attributed to the simplified shape of the single actuator used in the analytical model.

## 3. Finite-Element Analysis

**Figure 11:**
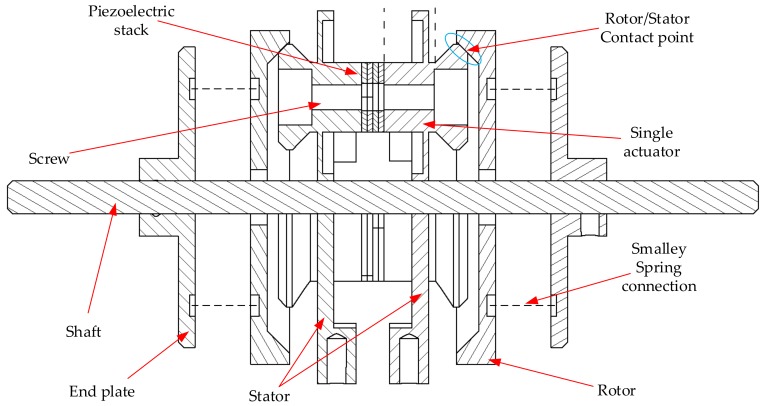
Cross-section of the full MPM structure.

**Figure 12:**
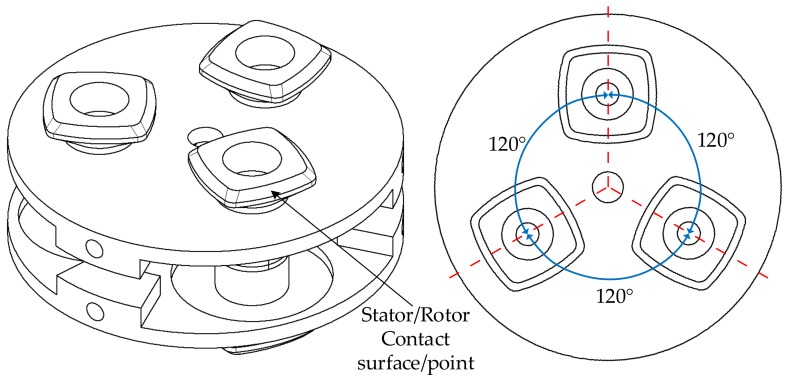
The structure of the stator.

The full virtual model of the MPM is presented in Figure 11, while the model of the stator is shown in Figure 12. The finite-element analysis (FEA) in an Ansys environment was used to determine the deformation characteristics and resonance frequencies of the MPM. The stator material was aluminium alloy with a mass density of 2800 kg/m${}^{3}$, a Young’s modulus of 7.17 ×$10^{10}$ N/m${}^{2}$, and a Poisson ratio of 0.32. The material of the ceramic ring was NCE81 PZT (provided by Noliac). Its main material properties are presented in Table 2.

**Figure 13:**
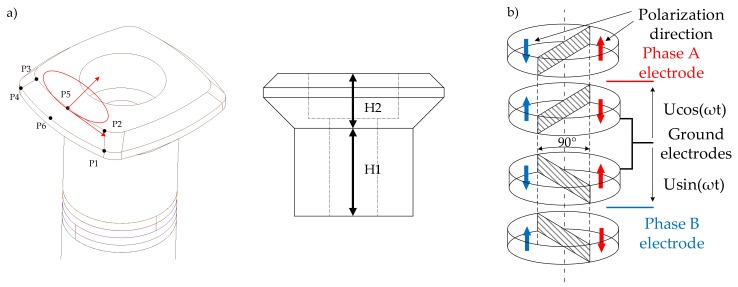
(**a**) Investigation points for static analysis and height investigation parameters; (**b**) polarization and arrangement of the PZT ceramic rings in a single actuator of the MPM.

Two types of analysis were conducted: modal simulation to obtain dynamic performance of the structure, and static simulation to acquire the stress levels. Moreover, the stator’s dimensions were investigated as a parameter. The influence of H1 and H2 values was examined in terms of resonance frequency and amplitude of vibration changes (Figure 13a). The precision of the simulation was dependant on the mesh density and shape of the finite elements. It was crucial to get an equal shape of the elements. The mesh density was increased in critical areas of the MPM. The average amount of the nodes and elements for both static and modal simulations were 52,000 and 30,000, respectively.

### 3.1. Modal Simulation

**Figure 14:**
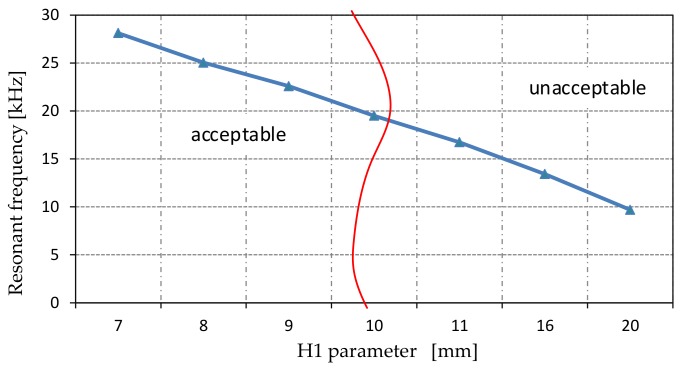
Frequency of bending mode of the stator as a function of the H1 parameter.

The main goal of a modal analysis was to obtain the resonance frequency of actuators above 20 kHz, which was in the ultrasonic range. Figure 14 illustrates the resonance frequency change in terms of height H1 of the actuator.

**Figure 15:**
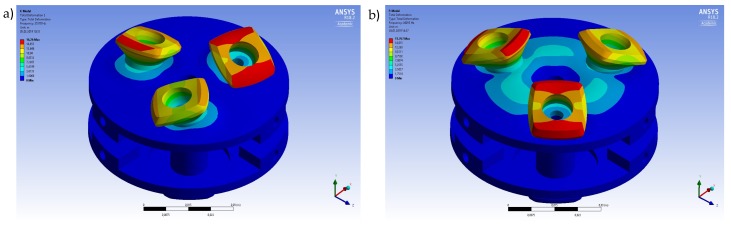
Modal simulation of the stator for two bending modes, 22.573 kHz and 22.575 kHz.

**Figure 16:**
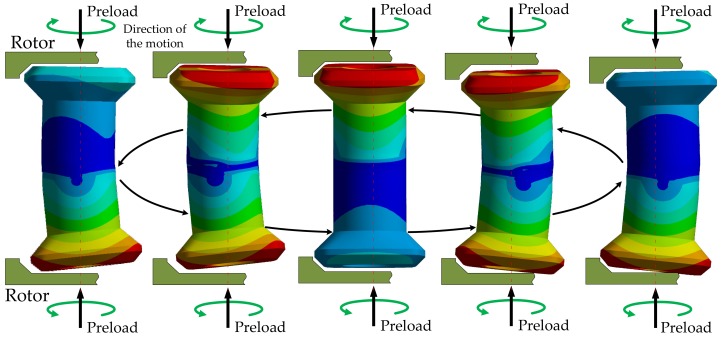
Work cycle of a single actuator in the proposed motor.

The results of the modal analysis have shown that several modes were observed in a 20 kHz–100 kHz frequency sweep range. However, only two frequencies were useful in terms of traveling wave generation. These frequencies corresponded to bending modes (Figure 15). The resonance frequencies of the two selected vibration bending modes must be nearly equivalent to ensure the proper performance of the motor (Figure 16). Other resonance frequencies were linked with respiration modes or with parasitic deformations of the armature.

As can be seen, not all of H1 parameters adapt to the MPM constrains. Results for the H1 parameter above 9 mm (around 23 kHz for 9 mm) were rejected from further analysis. Simulation results demonstrated that there were significant differences in resonance frequency corresponding to H1 changes, as expected. On the contrary, the H2 parameter did not have much influence on the frequency of bending modes, thus it was not considered.

### 3.2. Static Simulation

The purpose of the static analysis was to obtain the deformation level of the MPM’s actuators. To reduce the simulation time, a single actuator was analyzed. Figure 16 shows the operating principle of the MPM single actuator. The analysis was performed as a parametric study of deformation magnitudes as a function of actuator heights H1 and H2.

The sinusoidal excitation voltage was applied to the piezoelectric ceramic rings. During the simulations, the voltage amplitude was set to 200 V. Each ceramic was divided into halves and polarized along its thickness. The polarization directions of adjacent PZT ceramic rings were opposite. Moreover, two pairs of ceramics were rotated by 90${}^{\circ }$ to each other (Figure 13b). Depending on the presented step of the work cycle, half of the piezoelectric ceramic was either shrinking or extending. Finally, as a result of the appropriate synchronization, the travelling wave was generated on the surface.

**Figure 17:**
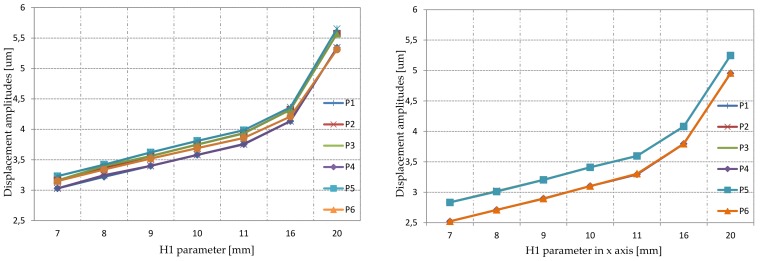
Deformation amplitudes for the selected analysis points as a function of parameter H1: Total displacement and displacements in the perpendicular axis of the actuator.

**Figure 18:**
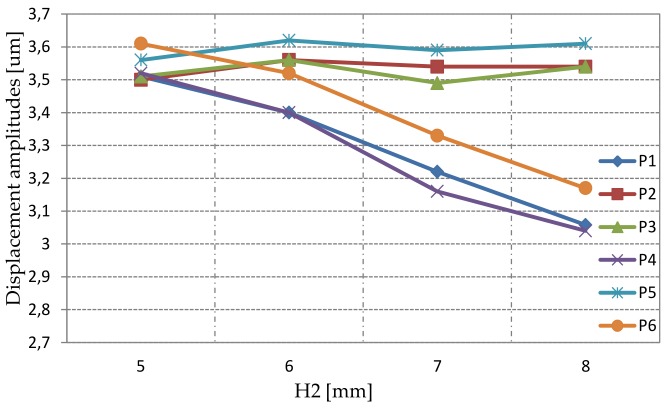
Deformation amplitudes for the selected analysis points as a function of parameter H2: Total displacement.

The deformation amplitude was investigated in six different points (Figure 13a) as a function of H1 and H2 parameters. The simulation results are shown in Figure 17. As expected, the deformation increased linearly in terms of height change. Moreover, all points had comparable displacement amplitudes. According to modal and static simulation results, the optimal H1 value was 9 mm. An additional analysis in terms of changing H2 and constant H1 (H1 = 9 mm) was performed (Figure 18). The simulation results showed a constant value of deformation in points P2, P3, and P5 while H2 was increasing. However, the deformation at P1, P4, and P6 was decreasing. The H2 equal to 6 mm was chosen due to the most effective inclination angle.

**Figure 19:**
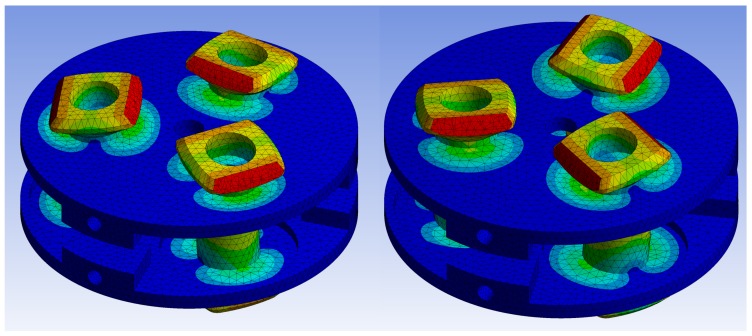
Stator static simulation illustrating two steps of the work cycle.

In order to validate the motor performance for selected H1 and H2 values, the complete structure of the stator was simulated. Figure 19 illustrates two chosen steps of a work cycle. These steps correspond to steps 2 and 4 from Figure 16, respectively.

Based on the dimensions of the initial structure and the FEA results, the final structural dimensions of MPM were as follows: the diameter of the stator was equal to 50 mm, and each actuator, a diameter of 12.5 mm. The surface around the actuator was 1 mm thick, and the diameter of the surface around the actuator was 8 mm. The external diameter was 60 mm, the internal diameter was 10 mm, and the inclination angle was 45${}^{\circ }$. The ceramics had the following dimensions: external diameter 12.5 mm; internal diameter 5 mm; and thickness 1 mm.

## 4. Experimental Analysis

**Figure 20:**
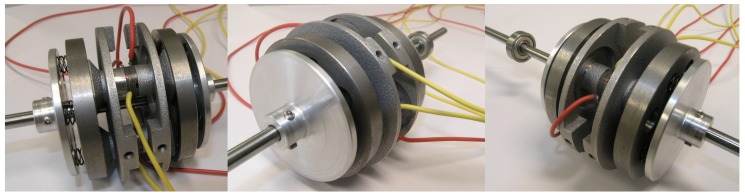
Full assembly of the prototype of MPM.

The prototype of the proposed piezoelectric motor is shown in Figure 20. The counter-mass was manufactured using additive manufacturing (3D printer). The material used for the production of the counter-mass was aluminum, due to its high resonance frequencies and mechanical losses on a satisfactory level. The rotor was manufactured using steel. The rotational motion of the rotor was transmitted to the shaft by “Smalley” springs and two plates.

**Figure 21:**
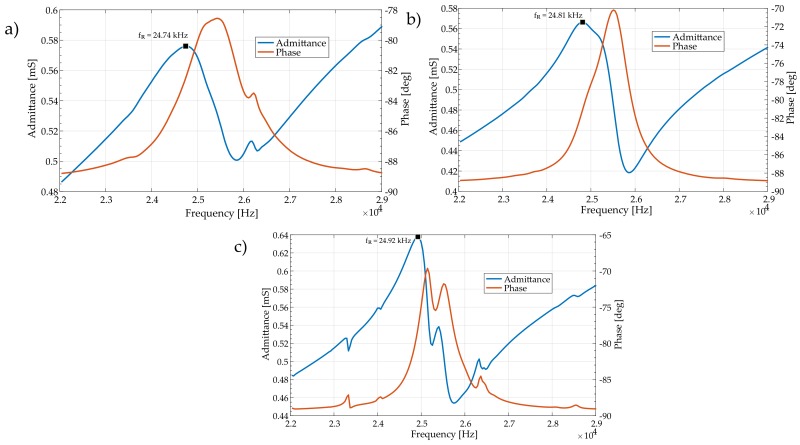
Admittance (amplitude and phase) measured near the resonance frequency: (**a**) first (**b**) second (**c**) third actuator.

The first stage of experimental analysis consisted of resonance frequency measurements for all three actuators. The Keysight E4990A Impedance Analyzer was used for this purpose. An essential aspect of this stage was to obtain similar resonance frequencies for all three actuators. The measured frequencies were in the range of 24.74 to 24.92 kHz. Bode plots for all three actuators are presented in Figure 21.

**Figure 22:**
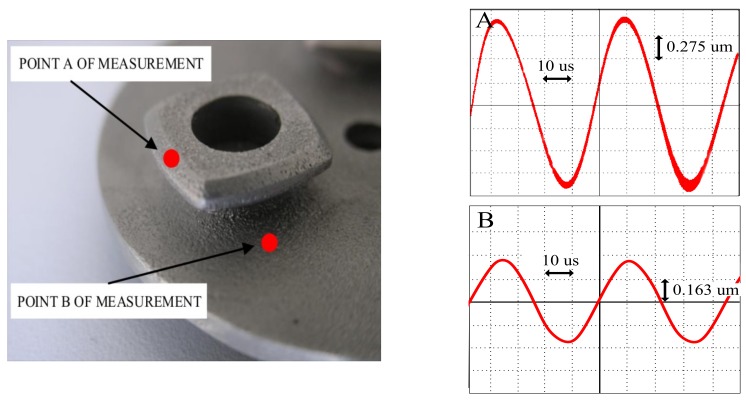
Points of measurement for the laser’s head and displacement amplitudes at points A and B (frequency of resonance f = 24.63 kHz).

The second stage of experimental analysis included a deformation measurement by a Polytec CLV laser vibrometer system. The surface of the stator was investigated. The measurement at point A was made on the surface which came into contact with the rotor. A measurement at point B was made on the stator armature, near the placement of the actuator. The measured displacements at points A and B are presented in Figure 22. The values of sinusoidal displacements at point A and B were 2.2 μm and 0.65 μm (peak-to-peak), respectively. The latter value was decreasing if the distance between point B and the actuator was increasing. In comparison with the FEM analysis results (3.5 μm and 0.7 μm respectively), the measured displacement values can be considered as satisfactory. Moreover, the measured displacement wave-forms were sinusoidal, as was required.

**Figure 23:**
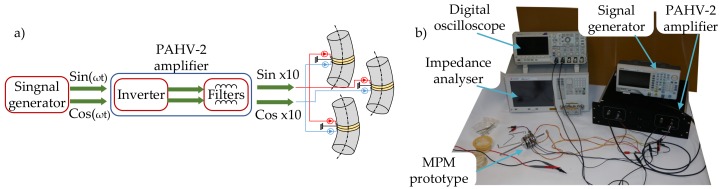
(**a**) A block diagram of the power amplification stage; (**b**) the experimental setup for performance testing.

**Figure 24:**
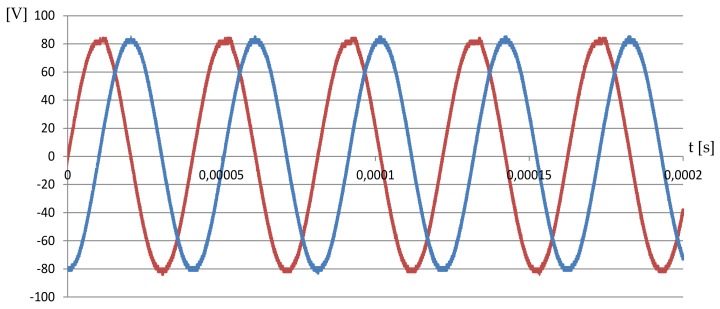
The voltage at the PAHV-2 power amplifier output during the mechanical parameters test.

**Figure 25:**
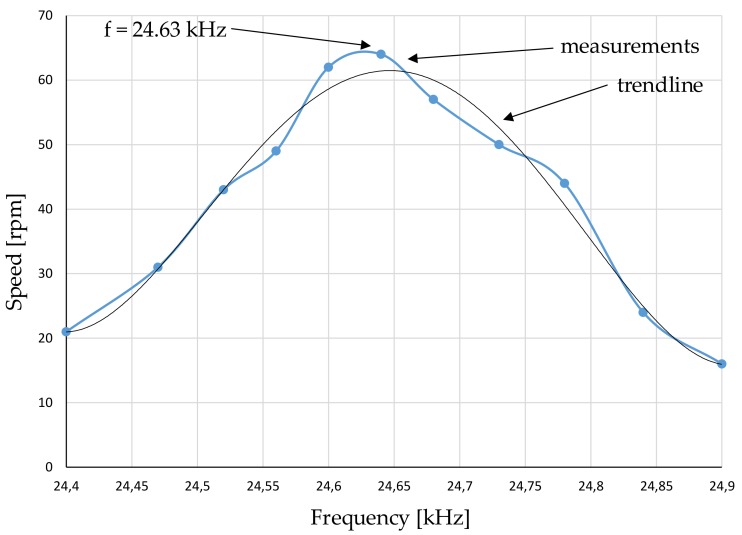
Rotary speed of MPM as a function of excitation frequency.

Finally, the mechanical parameters of speed and torque were measured (Figure 23). The power amplification stage (high-voltage linear amplifier PAHV-2 and signal generator) with two sinusoidal voltage sources was used (Figure 24). The voltage and frequency of the excitation signal were set to 164 $V_{P-P}$ and 24.63 kHz, respectively. With the above power supply limitations, the maximal measured blocking torque was 0.06 Nm. The influence of the frequency change on the rotary speed was measured by the digital, non-contact tachometer, while torque was measured using a known weight of 50 g attached to the shaft of MPM. The registered results are presented in Figure 25. The maximum rotary speed was 63 rpm. Higher rotary speed and torque can be achieved by increasing the output voltage of the power amplification stage or by using three stages tuned to the resonance frequencies of individual actuators.

## 5. Conclusions

In this paper, the concept of a novel piezoelectric motor, referred to as a ”multicell piezoelectric motor”, was developed and tested. The MPM prototype consists of the following parts: the stator built from two pairs of piezoelectric ceramics and counter-masses with three rotating-mode actuators, two rotors, special springs, two ending plates, and a shaft.

A finite element analysis was carried out in an Ansys environment to determine the resonance frequencies and displacements in counter-mass structures. The results of FEA studies have been compared with analytical model and experimental measurement results. The resonance frequencies results were 22.57 kHz, 24.90 kHz, and 24.81 kHz, respectively. The measured vibrations on the stator’s were 2.2 μm for an excitation voltage of 164 $V_{P-P}$. The optimum excitation frequency for pushing the rotors was 24.63 kHz—a balance between slight variations of resonance conditions for all three actuators. Nevertheless, the results have shown satisfactory accuracy and provided useful perspectives for further MPM study and design. The authors tried to find a compromise between blocking torque and maximum speed. The prototype obtained a peak no-load speed of 63 rpm, and the maximum blocking torque was 0.06 Nm.

Compared with recent piezoelectric motors, the MPM results are reasonable. In [34], the authors obtained a maximum speed of 366 rpm and 0.072 Nm with 350 $V_{P-P}$, where a 150 $V_{P-P}$ speed was around 50 rpm. The industrial solution, such as the Shinsei motor USR30-S3 [35], has 250 rpm and a rated torque of 0.05 Nm. However, this is a commercial and well-refined product.

In future research, the authors will focus on the improvement of efficiency and torque output of MPM. The idea is to increase the rotor and stator contact surface to achieve a greater effect of friction. Moreover, various stator shapes will be considered.

## Figures and Tables

**Table 1:** Physical quantities used in an analysis.

Quantity	Meaning	Unit
PZT	piezoelectric ceramic	-
M	counter-mass, mechanical equivalent of Inductance (L)	kg
K	screw spring constant, mechanical equivalent of the inverse of Capacitance (1/C)	N/m
$D_{M}$	damping factor, mechanical equivalent of Resistance (R)	Ns/m
v(t)	Voltage, electrical equivalent of Force	V
i(t)	current, electrical equivalent of Velocity	A
$F_{l}\left(t\right)$	load force	N
$F_{p}\left(t\right)$	force generated by PZT	N
u(t), $\dot{u}\left(t\right)$	position and speed of mass	m, m/s
*N*	electromechanical coupling ratio equal to $hC_{0}$	N/V
$A_{c}$	area of the flat surface of the PZT	m${}^{2}$
ρ	density of medium	kg/m${}^{3}$
*c*	compliance, mechanical equivalent of Capacitance (C)	m/N
ω	angular frequency	rad/s
α, β	coefficients depending on the boundary conditions	-
${\dot{U}}_{0}$, ${\dot{U}}_{L_{N}}$	vibration speed at the extremities of the analyzed element	rad/s
$F_{0}$, $F_{L_{N}}$	force at the extremities of the analyzed element	N
$Z_{C}$	characteristic impedance of the medium	kg/s
*T*	stress	N/m${}^{2}$
*E*	electric field	V/m
*D*	electric displacement	C/m${}^{2}$
$S^{D}$, $S^{E}$	strain under constant D and E, respectively	-
$c^{D}$, $c^{E}$	stiffness under constant D and E, respectively	N/m${}^{2}$
ϵ	relative permittivity	-
$d^{t}$, *g*	piezoelectric coefficients	C/N, m${}^{2}$/C
*h*, *e*		N/C, C/m${}^{2}$

**Table 2:** Properties of the piezoelectric ceramic, NCE81.

Parameter	Symbol	Model Value
Relative dielectric constant	$\varepsilon_{33}^{T}/\varepsilon_{0}$	1020
	$k_{31}$	0.30
Electromech. coupling factors	$k_{33}$	0.69
	$k_{31}$	0.47
Piezoelectric charge constant	$d_{31}$	−108
	$d_{33}$	−269
Quality factor	$Q_{M}$	1400
Density	ρ	7730
